# Occupational risk factors for chronic kidney disease in Andhra Pradesh: ‘Uddanam Nephropathy’

**DOI:** 10.1080/0886022X.2020.1824924

**Published:** 2020-10-12

**Authors:** Youssef M. K. Farag, Kuyilan Karai Subramanian, Vikrum A. Singh, Ravi Raju Tatapudi, Ajay K. Singh

**Affiliations:** a Department of Epidemiology, Johns Hopkins Bloomberg School of Public Health, Baltimore, MD, USA; b Renal Division, Brigham and Women’s Hospital, Boston, MA, USA; c Harvard Medical School, Boston, MA, USA; d Andhra Medical College & King George Hospital, Visakhapatnam, India

**Keywords:** chronic kidney disease, occupational risk factors, farmers, Andhra Pradesh, India, Uddanam Nephropathy

## Abstract

**Background:**

CKD of unknown etiology (CKDu) has been reported in several countries including India. We previously showed a prevalence of CKD in India to be 17.2% and we found a CKD epidemic in Andhra Pradesh (AP) to be 46.8%. We conducted this study to further explore the unexplained CKD epidemic in AP.

**Methods:**

We recruited 1201 adult participants through systematic random sampling from eight administrative divisions. Demographic, medical, and detailed occupational history was collected. Anthropometric measurements and blood pressure were taken and blood and urine samples were collected. Poisson regression model was used to identify potential predictors for CKD.

**Results:**

We analyzed data for 1184 individuals with mean age of 44.6 ± 14.0 years, of whom 44% were male. Prevalence of CKD was 32.2%. Working as a farmer had 20% more prevalence of CKD compared to non-farmers in the fully adjusted model (PR 1.2, 95% CI 1.01–1.42). Age, alcohol consumption, and chewing tobacco were also independent predictors of CKD. Gender, hypertension, and diabetes were not associated with CKD.

**Conclusions:**

The prevalence of CKD in AP is 32.2%. Occupational exposure among farmers could play a potential role in this epidemic. Large longitudinal epidemiologic research studies are needed to trace the causes of this problem.

## Introduction

Chronic kidney disease (CKD) is considered as an under-recognized epidemic in developing countries. Diabetes mellitus and hypertension have remained as traditional risk factors for developing CKD, while other emerging risk factors have not been studied extensively [1,2].

Screening and Early Evaluation of Kidney disease (SEEK) study was conducted in 2005 in India to evaluate the prevalence of CKD in India [3]. Over a period of 24 months, SEEK-India included 5588 participants from 53 screening camps in 12 cities across India representing almost all Indian regions, and there was an equal representation of urban and rural areas. We observed a 17.2% prevalence of CKD that was 2.5 times higher in urban vs. rural areas, with substantial center-to-center variation. Participants from Andhra Pradesh (AP) in the SEEK-India study showed 46.8% prevalence of CKD. However, the small sample size of the participants from AP showed that more extensive study is needed to determine the true prevalence of CKD in the ‘CKD-endemic’ areas.

Over the past 30 years, there has been a steady increase in the burden of CKD of unknown etiology (CKDu) in several countries, such as El Salvador [4–6], Nicaragua [7,8], Sir Lanka [9,10], and Egypt [11]. Several environmental and occupational risk factors have been proposed to contribute to the development of CKDu in the absence of diabetes and hypertension in rural populations of young male farmworkers.

The primary objective of the SEEK-Andhra study, a sub-study of the SEEK that was launched in 2010, is to better estimate the prevalence and risk factors of CKD in the north eastern province of AP with specific emphasis on nontraditional and potential occupational exposures.

## Methods

### Study design and sampling

The Screening and Early Evaluation of Kidney Disease-Andhra Pradesh (SEEK-Andhra) is a cross-sectional study that was conducted in April and May 2011 in the villages of Srikakulam district in AP. A systematic random sampling technique was conducted to assemble the study sample. According to the 2010 census, the total population of Srikakulam district was approximately 2.8 million. The district was divided into 16 mandals (administrative divisions) out of which eight mandals were randomly selected for the SEEK-Andhra study.

### Study population

All the subjects who were ≥18 years of age were included in the study. Those subjects who were unable to give the consent, those who were unwilling, bed-ridden patients, and pregnant women were excluded from the study.

We designed our study to have 95% power to detect at least 25% higher prevalence in AP than the national CKD prevalence that we previously reported of 17.2% [3]. Type-I error (*α*) was set at 0.05. This yielded 1084 individuals. To account for potentially missing data of approximately ∼10% after the conclusion of the project, we planned to recruit 1200 participants. To ensure equal representation of the population from all the eight mandals, a total of 150 subjects per mandal were selected for the study. Domiciliary screening was conducted and a maximum of two adult individuals from each household who met the eligibility criteria were selected for the study. A total of 1201 subjects were selected by systematic random sampling.

**Figure 1.**
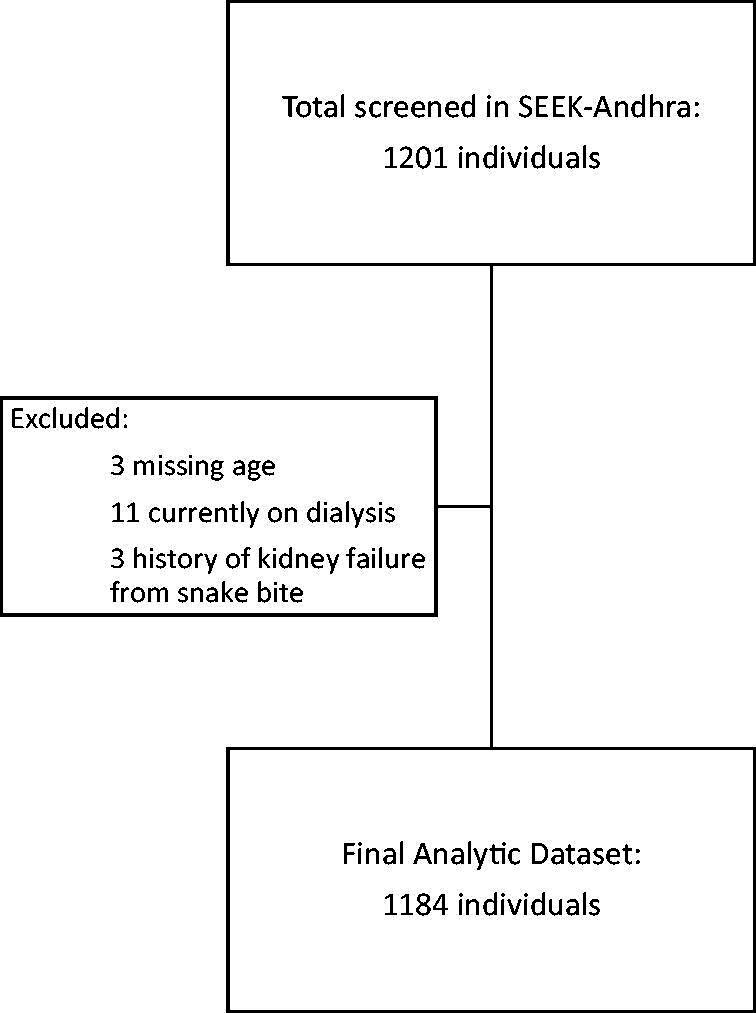
Flowchart of SEEK-Andhra Study participants.

The purpose of the study was explained to the subjects and informed consent was obtained in the regional language by the trained district health staff. Analytic dataset was constructed based on complete case analysis approach. It consisted of 1184 individuals who had non-missing values on age, gender, serum creatinine, and urine dipstick protein (98.6% of the study population). A participant flow diagram is outlined in Figure 1.

### Laboratory assays

Blood and urine were collected at the site by trained phlebotomists. Serum creatinine, blood urea nitrogen, random glucose, sodium, potassium, chlorides, and bicarbonates, hemoglobin were measured using Hitachi Roche analyzer in a NABL (National Accreditation Board for Testing and Calibration Laboratories) accredited lab. Serum creatinine was measured using Jaffe Colorimetric method. Urine collected in a 15 mL container was analyzed for urine albumin and glucose measured using visually read dipsticks (Bayer Multistix 10 SG). Urine routine analysis and urine microscopy (qualitative and quantitative analysis) were also carried out.

### Exposure variables assessment

Specially designed CRFs were administered by the trained interns in the local language. The questionnaire included details on the socio-demographic data, a detailed occupational history, data on exposure to pesticides or any harmful chemicals related to their occupation, detailed data on food and drinking water sources, symptoms of diabetes, hypertension or kidney disease, past/family history of hypertension and diabetes, treatment for diabetes and hypertension, medication history, analgesic usage and history of tobacco and alcohol usage. Anthropometric measurements including height, waist, and hip circumference were measured.

Weight was measured in light clothes without footwear. Body mass index (BMI) was calculated as weight in kilograms divided by height in meters squared. Waist circumference was used to assess the body fat distribution and was measured using a non-stretchable tape to the nearest 0.1 cm at the mid-point between coastal margin and iliac crest. Hip circumference was measured at the level of the greater trochanters to the nearest 0.1 cm (widest portion of the hip) by a measuring tape, while the subject was standing with their arms by their side and feet together. A waist circumference ≥90 cm in males and ≥80 cm in females was used as a cutoff for central/abdominal obesity.

All participants underwent two blood pressure measurements at an interval of 5 min and the average of the two readings was finally recorded. Blood pressure was measured using guidelines from American Heart Association. Hypertension was defined as a systolic blood pressure ≥140 mmHg, a diastolic blood pressure ≥90 mmHg, the use of medications for high blood pressure, or a self-reported diagnosis of high blood pressure. Diabetes was defined as a random blood glucose ≥200 mg/dL, the use of anti-diabetic medications, or a self-reported diagnosis of diabetes mellitus.

### Outcome variable assessment

CKD was defined following the KDIGO 2012 Clinical Practice Guideline for the Evaluation and Management of Chronic Kidney Disease [12] of impaired kidney function or kidney damage. Impaired kidney function was defined as an estimated glomerular filtration rate (eGFR) less than 60 mL/min/1.73 m^2^ using the CKD-EPI equation [13]. Kidney damage was defined as proteinuria; total urine protein to urine creatinine of more than 150 g/mg, or positive urine protein dipstick (+1, +2, and +3), or hematuria; positive red blood cells on urine microscopy or positive urine dipstick for red blood cells. Females who reported current menstruation and had hematuria were labeled as not having hematuria.

### Ethics approval

The study was approved by both the Institutional Review Board (IRB) of Partners Healthcare and the local institutional ethics committee of Andhra Medical College in Andhra Pradesh.

### Statistical analysis

The data collected from the subjects were checked for accuracy on the site, and were then entered in Microsoft Excel spreadsheets. The data were imported for further data management and statistical analysis using Stata 14 (StataCorp, 2015, Stata Statistical Software: Release 14, StataCorp LP, College Station, TX).

Descriptive summary statistics of the baseline demographic, clinical, and occupational variables were presented for the total study population and by the absence or presence of CKD. Continuous variables were presented using means and standard deviations (SDs), or medians and first and third quartiles (Q1, Q3) , as appropriate, and we compared the two outcome groups using Student’s *t*-tests for normally distributed continuous variables, and Mann–Whitney’s *U* test for non-normally distributed variables. Categorical variables were presented using frequencies and proportions, and we compared the two outcome groups using the Chi-squared test. If there was a cell in cross tabulating the categorical variables with expected frequency less than 5, we calculated Fisher’s exact test’s *p* value.

To identify the predictors for CKD, we decided to use Poisson regression to estimate the prevalence ratios and the corresponding 95% confidence intervals (CIs). Given the prevalent CKD outcome (>15%), Poisson regression was a more appropriate model since the use of the logistic regression was shown to overestimate the association between the exposure and the prevalent binary outcome [14]. We constructed crude, simple, and progressively adjusted multivariable Poisson regression models with robust variance estimation. Model 1 was the crude unadjusted model. Model 2 was adjusted for age and gender. Model 3 was further adjusted for lifestyle variables: smoking, tobacco chewing, alcohol drinking, exercise, and consumption of vegetarian diet. Model 4 is our full model that was adjusted for all the variables in models 2 and 3, and further adjusted for waist–hip ratio, hypertension, diabetes, and self-reported cardiovascular disease. For all the statistical tests, a two-sided *p* value of less than .05 was considered statistically significant.

## Results

### Demographic and clinical characteristics

The mean ± SD age of the study population of SEEK-Andhra 44.6 ± 14.0 years, of whom 44% were male. Most of the study participants lived in the region for over 20 years (83.3%) and made 2000–4999 rupees/month. The majority also reported being never smoker, never chewed tobacco, and never drank alcohol. Furthermore, 45% of them were hypertensive and 22% were diabetic. Other summary statistics are presented in Table 1. Individuals with CKD were more likely to be older, males, illiterate, stayed longer in the region, present or past smoker, and current alcohol drinker. They were also more likely to be normal or underweight and have hypertension. Detailed comparison between individuals with CKD and those without is presented in Table 1.

**Table 1.** Baseline demographic and clinical characteristics of the total SEEK-Andhra Study Participants, and by CKD status (bold denotes statistical significance).

		CKD		*p* Value
	Total study population	No	Yes	
*N*	1184	803	381	
Age, years, mean ± SD	44.6 ± 14.0	41.7 ± 13.3	50.7 ± 13.5	<.001
Gender				<.001
Male	526 (44.4%)	327 (40.7%)	199 (52.2%)	
Female	658 (55.6%)	476 (59.3%)	182 (47.8%)	
Family income				.52
<2000 rupees/month	235 (19.8%)	151 (18.8%)	84 (22.0%)	
2000–4999 rupees/month	614 (51.9%)	416 (51.8%)	198 (52.0%)	
5000–9999 rupees/month	236 (19.9%)	163 (20.3%)	73 (19.2%)	
10,000–19,999 rupees/month	64 (5.4%)	48 (6.0%)	16 (4.2%)	
≥20,000 rupees/month	35 (3.0%)	25 (3.1%)	10 (2.6%)	
Education				**<.001**
Illiterate	504 (42.6%)	292 (36.4%)	212 (55.6%)	
8th grade or less	354 (29.9%)	262 (32.6%)	92 (24.1%)	
9–12th grade	251 (21.2%)	189 (23.5%)	62 (16.3%)	
Some college	9 (0.8%)	6 (0.7%)	3 (0.8%)	
Graduate degree	55 (4.6%)	45 (5.6%)	10 (2.6%)	
Postgraduate/masters/doctorate	11 (0.9%)	9 (1.1%)	2 (0.5%)	
Duration of stay in the region				**.003**
0–10 years	86 (7.3%)	68 (8.5%)	18 (4.7%)	
11–20 years	112 (9.5%)	87 (10.8%)	25 (6.6%)	
more than 20 years	986 (83.3%)	648 (80.7%)	338 (88.7%)	
Vegetarian	173 (14.6%)	122 (15.2%)	51 (13.4%)	.41
Smoking status				**<.001**
Never	795 (74.6%)	575 (79.9%)	220 (63.6%)	
Past	93 (8.7%)	49 (6.8%)	44 (12.7%)	
Present	178 (16.7%)	96 (13.3%)	82 (23.7%)	
Chew tobacco				**<.001**
Never	1048 (88.5%)	732 (91.2%)	316 (82.9%)	
Past	29 (2.4%)	20 (2.5%)	9 (2.4%)	
Present	107 (9.0%)	51 (6.4%)	56 (14.7%)	
Alcohol drinking				**<.001**
Never	917 (77.4%)	664 (82.7%)	253 (66.4%)	
Past	52 (4.4%)	32 (4.0%)	20 (5.2%)	
Present	215 (18.2%)	107 (13.3%)	108 (28.3%)	
Height, cm, mean ± SD	156.0 ± 9.0	156.0 ± 9.2	156.0 ± 8.6	.90
Weight, kg, mean ± SD	54.7 ± 15.6	55.5 ± 12.8	53.1 ± 20.2	**.011**
Body mass index, kg/m^2^, median (Q1, Q3)	21.8 (18.9, 25.1)	22.1 (19.2, 25.5)	21.0 (18.3, 23.7)	**<.001**
Waist to hip ratio, mean ± SD	0.9 ± 0.1	0.88 ± 0.01	0.9 ± 0.08	**.004**
Obesity				**<.001**
Normal	638 (53.9%)	426 (53.1%)	212 (55.6%)	
Underweight	247 (20.9%)	144 (18.0%)	103 (27.0%)	
Overweight	236 (19.9%)	182 (22.7%)	54 (14.2%)	
Obese	48 (4.1%)	38 (4.7%)	10 (2.6%)	
Morbidly obese	14 (1.2%)	12 (1.5%)	2 (0.5%)	
Mean systolic blood pressure, mmHg, mean ± SD	129.3 ± 20.3	126.8 ± 17.6	134.8 ± 24.0	**<.001**
Mean diastolic blood pressure, mmHg, mean ± SD	82.8 ± 11.7	81.8 ± 10.5	85.1 ± 13.7	**<.001**
Hypertension	539 (45.5%)	343 (42.7%)	196 (51.4%)	**.005**
Random blood glucose, mg/dL, mean ± SD	110.8 ± 45.8	106.3 ± 34.4	120.0 ± 62.2	**<.001**
Diabetes	262 (22.1%)	175 (21.8%)	87 (22.8%)	.69
Hemoglobin, g/dL, mean ± SD	12.1 ± 2.2	12.3 ± 2.1	11.6 ± 2.4	**<.001**
Serum creatinine, mg/dL, median (Q1, Q3)	0.8 (0.6, 1.0)	0.7 (0.6, 0.9)	0.9 (0.7, 1.3)	**<.001**
Urine protein to creatinine ratio, median (Q1, Q3)	73.0 (53.8, 110.2)	63.6 (50.0, 84.7)	130.8 (73.9, 222.8)	**<.001**
Self-reported kidney stones	37 (3.1%)	21 (2.6%)	16 (4.2%)	.14
Self-reported cardiovascular disease	168 (14.2%)	107 (13.3%)	61 (16%)	.2

### Occupational characteristics

Approximately, 44% of the total study population were working as farmers, and 51% had CKD. Individuals with CKD were more likely to wear personal protective equipment and work as farmers in rice cultivation. Other details on the occupational characteristics of the study population are presented in Table 2.

**Table 2.** Occupational characteristics and exposures of the total SEEK-Andhra Study Participants, and by CKD status (bold denotes statistical significance).

	Total study population	CKD		*p* Value
		No	Yes	
*N*	1184	803	381	
Farmer/agriculturist	523 (44.2%)	328 (40.8%)	195 (51.2%)	**<.001**
Wear personal protective equipment	89 (7.5%)	47 (5.9%)	42 (11.0%)	**.002**
Farmer in coconut plantations	151 (12.8%)	101 (12.6%)	50 (13.1%)	.79
Farmer in cashew nut plantations	219 (18.5%)	139 (17.3%)	80 (21.0%)	.13
Farmer in rice cultivation	374 (31.6%)	234 (29.1%)	140 (36.7%)	**.009**
Farmer in vegetable cultivation	43 (3.6%)	26 (3.2%)	17 (4.5%)	.29
Farmer in fruit cultivation	28 (2.4%)	17 (2.1%)	11 (2.9%)	.42
Spray pesticides	149 (12.6%)	94 (11.7%)	55 (14.4%)	.19
Use fertilizers	176 (14.9%)	113 (14.1%)	63 (16.5%)	.27
Mix other products with pesticides	91 (7.7%)	55 (6.8%)	36 (9.4%)	.12
Store pesticides at home	60 (5.1%)	41 (5.1%)	19 (5.0%)	.93
Wash clothes from pesticides				**.003**
No	1007 (85.1%)	700 (87.2%)	307 (80.6%)	
Mixed with family wash	47 (4.0%)	24 (3.0%)	23 (6.0%)	
Soaked separately, then mixed with family wash	27 (2.3%)	18 (2.2%)	9 (2.4%)	
Washed separately in family machine	37 (3.1%)	27 (3.4%)	10 (2.6%)	
Others	66 (5.6%)	34 (4.2%)	32 (8.4%)	
Clean self from pesticides				.11
No	1007 (85.1%)	697 (86.8%)	310 (81.4%)	
Wash exposed parts immediately with soap and water	48 (4.1%)	29 (3.6%)	19 (5.0%)	
Wash exposed parts with water only	21 (1.8%)	13 (1.6%)	8 (2.1%)	
Take whole body wash immediately	108 (9.1%)	64 (8.0%)	44 (11.5%)	
Factory worker	5 (0.4%)	4 (0.5%)	1 (0.3%)	1.00
Work in fishing	19 (1.6%)	13 (1.6%)	6 (1.6%)	.95

### Prevalence of CKD

**Figure 2.**
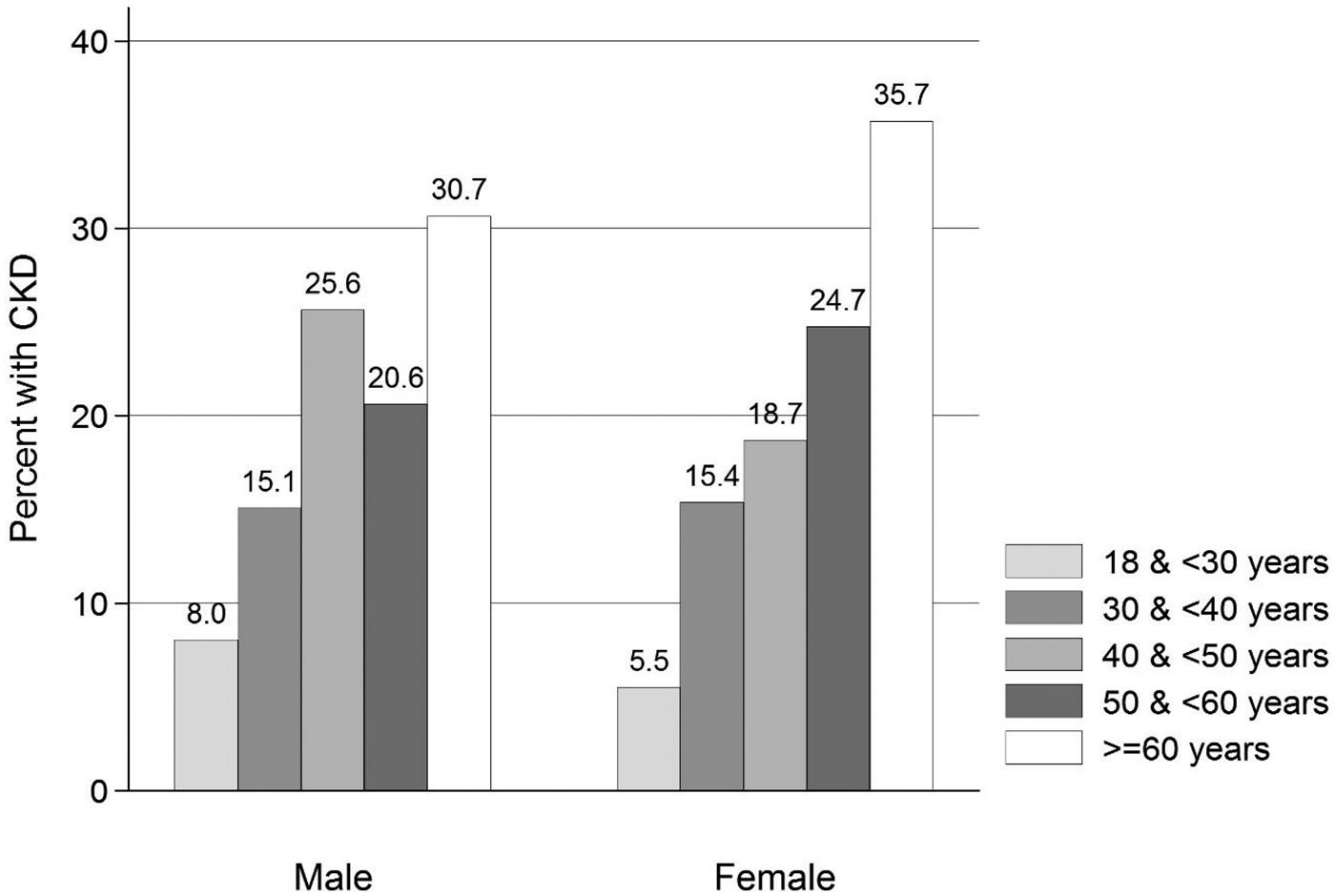
Prevalence of CKD in the SEEK-Andhra Study by gender and age categories.

**Figure 3.**
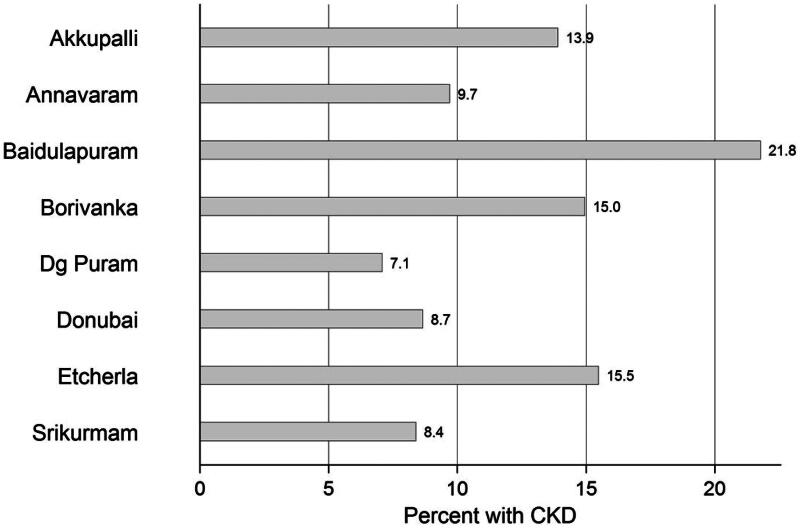
Prevalence of CKD in the SEEK-Andhra Study by primary health centers (PHC).

Using the KDIGO guidelines, CKD was present in 381 individuals (32.2%), half of them were males. Impaired kidney function (eGFR < 60 mL/min/1.73 m^2^) was found in 9.2%, while the prevalence of proteinuria was 15.5%, and hematuria was found in 20.2%. There was increasing prevalence of CKD with increasing age in both males and females. Eight percent of the males in the age category of 18 and less than 30 years had CKD, and 5.5% of females had CKD in the same age category. The highest prevalence of CKD was in the age category of ≥60 years, 30.7% of males and 35.7% of females (Figure 2). The prevalence of CKD in the different primary health centers ranged from 7.1% in Dg Puram to 21.8% in Baidulapuram (Figure 3).

### Risk factors for CKD

**Figure 4.**
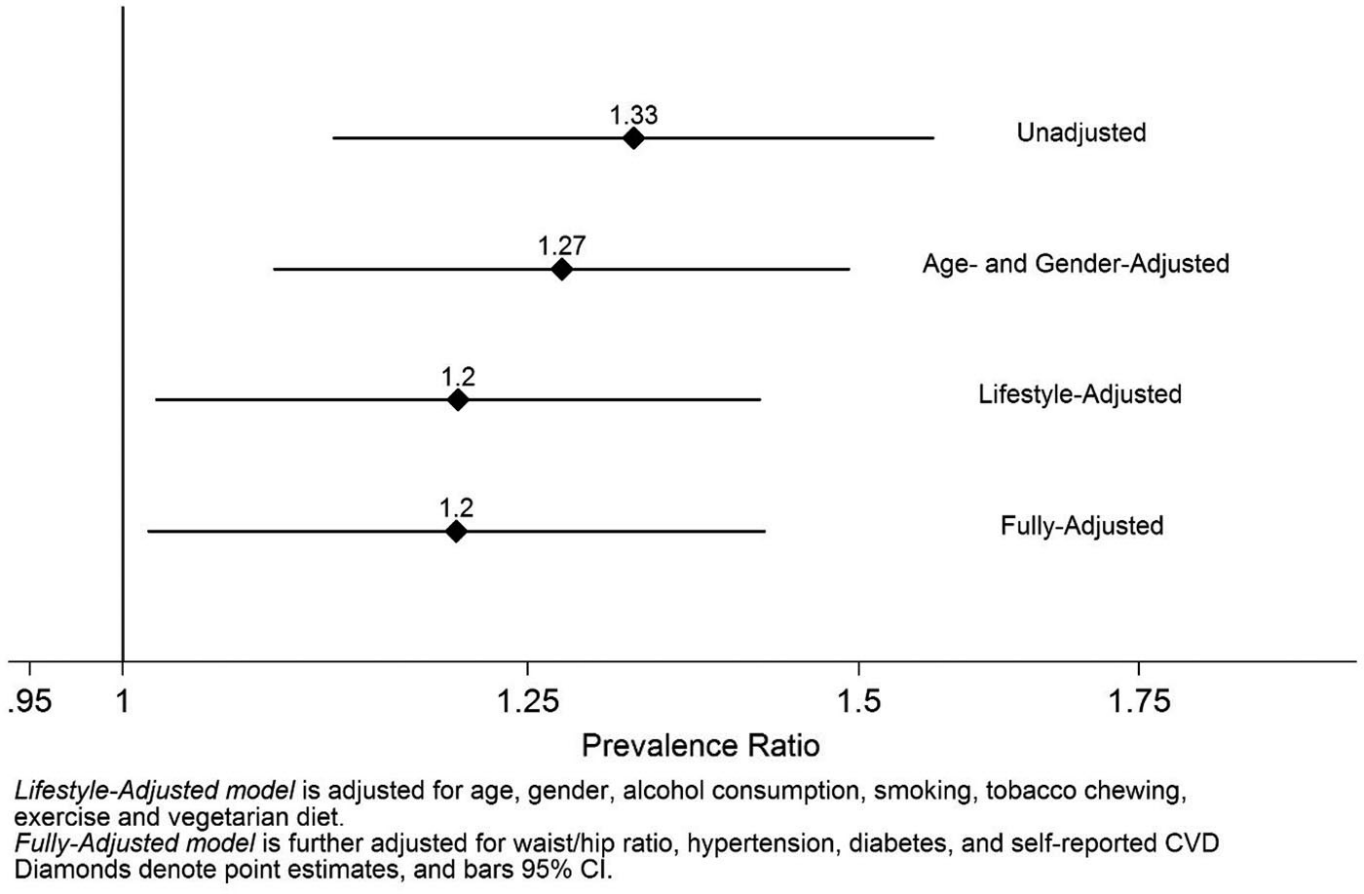
Prevalence ratios (95% confidence intervals) for CKD comparing farmers and non-farmers in the SEEK-Andhra Study.

The unadjusted and adjusted PRs (95% CI) of prevalent CKD according to demographic and medical risk factors are presented in Table 3. In model 1, which is unadjusted, several risk factors were associated with CKD in our study population. Age in years (PR 1.36, 95% CI 1.29–1.44), present alcohol consumption (PR 1.82, 95% CI 1.54–2.16), current smoking (PR 1.66, 95% CI 1.37–2.02), present chewing tobacco (PR 1.74, 95% CI 1.42–2.13), WHR (PR 4.38, 95% CI 1.7–11.25), and hypertension (PR 1.27, 95% CI 1.07–1.5) were highly associated with CKD. However, only age, alcohol consumption, and chewing tobacco were attenuated but remained statistically significant after multivariable adjustment. Females had statistically significant 27% less prevalence of CKD compared to men 0.73 (0.62–0.86). However, this association reached the null after progressive adjustment for risk factors in models 2, 3, and 4. Working as a farmer had 33% more prevalence of CKD compared to non-farmers (PR 1.33, 95% CI 1.12–1.56), and association was slightly attenuated to 20% but maintained statistical significance (PR 1.2, 95% CI 1.01–1.42) (Figure 4). We also explored occupational practices as potential risk factors for CKD. Those who are working as farmers in rice cultivation showed 20% statistically significant more prevalence of CKD than those who are not (PR 1.2, 95% CI 1.01–1.43). Detailed results are shown in Table 4.

**Table 3.** Prevalence ratios (95% confidence intervals) for CKD by demographic and medical risk factors (bold denotes statistical significance, WHR: waist/hip ratio, CVD: cardiovascular disease).

	Model 1	Model 2	Model 3	Model 4
Farmers				
No	1.00 (reference)	1.00 (reference)	1.00 (reference)	1.00 (reference)
Yes	**1.33 (1.12–1.56)**	**1.27 (1.09–1.49)**	**1.2 (1.02–1.42)**	**1.2 (1.01–1.42)**
Age (for every 10 years)	**1.36 (1.29–1.44)**	**1.35 (1.27–1.42)**	**1.32 (1.24–1.4)**	**1.31 (1.23–1.39)**
Gender				
Males	1.00 (reference)	1.00 (reference)	1.00 (reference)	1.00 (reference)
Females	**0.73 (0.62–0.86)**	**0.8 (0.68–0.94)**	0.97 (0.77–1.22)	0.99 (0.78–1.25)
Alcohol consumption				
Never	1.00 (reference)	1.00 (reference)	1.00 (reference)	1.00 (reference)
Past	1.39 (0.97–2)	1.24 (0.85–1.8)	0.96 (0.62–1.48)	0.92 (0.59–1.43)
Present	**1.82 (1.54–2.16)**	**1.7 (1.35–2.13)**	**1.48 (1.16–1.88)**	**1.48 (1.16–1.88)**
Smoking				
Never	1.00 (reference)	1.00 (reference)	1.00 (reference)	1.00 (reference)
Past	**1.71 (1.34–2.18)**	1.19 (0.93–1.52)	1.15 (0.89–1.48)	1.14 (0.88–1.46)
Present	**1.66 (1.37–2.02)**	1.21 (0.99–1.48)	1.09 (0.89–1.35)	1.08 (0.88–1.34)
Chewing tobacco				
Never	1.00 (reference)	1.00 (reference)	1.00 (reference)	1.00 (reference)
Past	1.03 (0.59–1.78)	1.02 (0.6–1.72)	1.06 (0.64–1.75)	1.06 (0.64–1.76)
Present	**1.74 (1.42–2.13)**	**1.52 (1.23–1.88)**	**1.34 (1.08–1.67)**	**1.33 (1.07–1.65)**
Regular exercise				
No	1.00 (reference)	1.00 (reference)	1.00 (reference)	1.00 (reference)
Yes	0.95 (0.69–1.32)	0.88 (0.64–1.21)	0.85 (0.6–1.22)	0.86 (0.6–1.22)
Vegetarian diet				
No	1.00 (reference)	1.00 (reference)	1.00 (reference)	1.00 (reference)
Yes	0.9 (0.71–1.16)	0.83 (0.66–1.05)	0.85 (0.67–1.09)	0.86 (0.68–1.1)
WHR (for every 1 cm)	**4.38 (1.7–11.25)**	0.81 (0.31–2.09)	1.08 (0.39–2.94)	1.09 (0.39–3.03)
Hypertension				
No	1.00 (reference)	1.00 (reference)	1.00 (reference)	1.00 (reference)
Yes	**1.27 (1.07–1.5)**	1.11 (0.94–1.3)	1.08 (0.91–1.27)	1.09 (0.91–1.3)
Diabetes				
No	1.00 (reference)	1.00 (reference)	1.00 (reference)	1.00 (reference)
Yes	1.04 (0.86–1.27)	0.95 (0.79–1.14)	0.99 (0.81–1.2)	0.97 (0.79–1.21)
Self-reported CVD				
No	1.00 (reference)	1.00 (reference)	1.00 (reference)	1.00 (reference)
Yes	1.15 (0.93–1.44)	1.07 (0.87–1.32)	1.13 (0.91–1.4)	1.1 (0.88–1.37)

**Table 4.** Prevalence ratios (95% confidence intervals) of CKD by occupational risk factors (bold denotes statistical significance).

	Fully adjusted model
Wear personal protective equipments	
No	1.00 (reference)
Yes	1.16 (0.9–1.49)
Farmer in coconut plantations	
No	1.00 (reference)
Yes	1.02 (0.79–1.32)
Farmer in cashew nut plantations	
No	1.00 (reference)
Yes	1.12 (0.91–1.39)
Farmer in rice cultivation	
No	1.00 (reference)
Yes	**1.2 (1.01–1.43)**
Farmer in vegetable cultivation	
No	1.00 (reference)
Yes	1.11 (0.75–1.63)
Farmer in fruit cultivation	
No	1.00 (reference)
Yes	1.04 (0.67–1.6)
Spray pesticides	
No	1.00 (reference)
Yes	0.99 (0.77–1.26)
Use fertilizers	
No	1.00 (reference)
Yes	0.99 (0.79–1.23)
Mix other products with pesticides	
No	1.00 (reference)
Yes	0.95 (0.71–1.27)
Store pesticides at home	
No	1.00 (reference)
Yes	0.83 (0.52–1.32)
Wash clothes from pesticides	
No	1.00 (reference)
Mixed with family wash	1.44 (1.09–1.9)
Soaked separately, then mixed with family wash	0.65 (0.3–1.4)
Washed separately in family machine	0.59 (0.32–1.11)
Others	1.26 (0.96–1.67)
Clean self from pesticides	
No	1.00 (reference)
Wash exposed parts immediately with soap and water	1.07 (0.74–1.53)
Wash exposed parts with water only	1.09 (0.64–1.84)
Take whole body wash immediately	1.05 (0.79–1.39)
Factory worker	
No	1.00 (reference)
Yes	0.76 (0.23–2.54)
Work in fishing	
No	1.00 (reference)
Yes	0.8 (0.34–1.91)

## Discussion

The main finding of this study is that the prevalence of CKD in the villages of Srikakulam district in AP was 32.2%. This observation of heightened prevalence of CKDu has been labeled as ‘Uddanam Nephropathy’. We also found that working as a farmer, age, alcohol consumption, and chewing tobacco were independent predictors of CKD after adjusting for potential confounders. Traditional risk factors of CKD like hypertension and diabetes were not association with CKD.

The CKD prevalence that we report here is much higher than what we previously reported for SEEK-India Study which was 17.2% [3]. This unexplained epidemic that we found in the AP subset of SEEK-India was the primary motivation to conduct the SEEK-Andhra, which was designed to overcome the primary limitations of this finding from SEEK-India, namely, larger sample size, domiciliary screening rather than screening camps, and detailed occupational history. Such high prevalence in AP was reported by other investigators as 15.2% for kidney damage, and 61% for reduced eGFR [15,16]. A study by Tatapudi et al. [17] in Uddanam showed that the prevalence of eGFR < 60 mL/min per 1.73 m^2^) was seen in 13.98% of their sample, while the prevalence of subjects having low eGFR and with proteinuria was 18.23%. The Indian CKD registry also reported that the highest number of CKD patients to be in the southern India [18]. However, prevalence estimates from other studies are based on smaller sample sizes, and registry estimates could be overestimated due to potential high reporting bias from an area that is already known to have high CKD prevalence. The coastal region of Saurashtra and non-coastal region of North Gujarat had CKD prevalence of 15.7% and 26%, respectively [19]. A recent population-based survey of Delhi and Chennai showed that the age standardized prevalence of CKD is 8.7% [20]. However, this study included an entirely urban population.

Hypertension, diabetes mellitus, and abdominal obesity are the traditional risk factors for CKD in India as shown in previous studies [3,18,19]. However, none of these were associated with CKD in the present study. A recent systematic review on CKDu summarizes the regional variation in the association between CKDu and potential risk factors [21]. Although these studies were heterogeneous in their exposure assessment, the most commonly associated risk factors for CKDu were male gender, age, agricultural occupation, family history of CKDu, snake bite, and heavy metal exposure. In our study, we excluded those individuals who reported previous snake bite, and we did not assess heavy metal exposure nor family history of CKDu. However, in our fully adjusted model, gender was not associated with CKDu. Both age and working as a farmer remained statistically significant.

Since the rural communities and farming are common themes in the areas where CKDu has been reported, pesticides have been proposed to be a potential cause of CKD [22]. Acute exposure to pesticides in rats [23], humans [24], and long-term low-level exposure in rats has been shown to be associated with kidney damage. Some biomarkers of toxicity induced by pesticides lead to glomerular inflammation, renal tubular epithelial cell swelling, and granular degeneration [25,26]. Many studies that sought to explore the association between CKD and various chemicals, including the present study, did not have biological biomarkers for these chemicals. Rather, self-reported use of or exposure to chemicals was used for exposure assessment, which is prone to recall bias. Such biological biomarkers could include blood and/or urine concentrations of pesticides, fertilizers, heavy metals like mercury and arsenic that are being used in the manufacturing of pesticides.

Furthermore, silica has been proposed as a potential risk factor for CKDu. Soil analysis that was conducted as part of the current study showed high levels of silica (unpublished data). Animal studies have shown that silica exposure to mice caused both inflammatory and fibrotic response in mice kidney [27]. A case-control study from the United States showed positive association between occupational exposure to silica and CKD in a dose–response fashion [28]. Another study from southern India in Canacona reported similar findings on the potential nephrotoxic role of silica [29].

Another potential source of exposure is contaminated drinking water. Reddy and Gunasekar [30] analyzed several drinking water samples in AP. They found that the levels of the major ions and trace elements in these samples were within the recommended levels set by regulatory bodies and guidelines, which make it unlikely that they have nephrotoxic impact. However, they did not measure the possible contamination of the drinking water by various organic and inorganic chemicals. In any case, exposure assessment in the ecosystem without biological biomarkers in human samples will not be enough to track down the source of the CKDu.

Our study has several limitations. First, the cross-sectional nature of the study has the inherent limitation of the inability to establish temporality in order to make causal inference. Subsequently, reverse causation is a possibility, but this may be unlikely since people with CKD will be sicker than other workers in the physically demanding job of farming. Second, kidney function was assessed using single measurement of serum creatinine. Third, we measured urine protein instead of urine albumin which might have overestimated the prevalence of CKD. Fourth, farming entails several sources of exposures that we have not tested due to limited funds.

Despite these limitations, our study has several strengths. First, we randomly selected the administrative divisions from which we will conduct our study. Second, we used systematic random sampling in the domiciliary house to house screening instead of the convenience sample of a screening camp. This could potentially decrease the high participation bias of sicker individuals, and subsequently a higher CKD prevalence in AP that we found in SEEK-India. Third, our sample size is considered the largest among all the published reports so far, which achieved its maximum designed power. Fourth, we collected detailed occupational history and explored its potential association with CKD. Fifth, we performed series of robust statistical analyses to control for potential confounders and calculate more precise effect estimates of each risk factor on CKD.

## Conclusions

In this large cross-sectional household screening, we found a very high prevalence of CKD of 32.2% among the rural communities of AP, specifically male farm workers. Traditional risk factors for CKD including hypertension and diabetes were not associated with CKD in the fully adjusted models. Working as farmer, increasing age, alcohol consumption, and chewing tobacco were independent risk factor for CKD. These findings urge the need for more carefully designed large longitudinal epidemiologic research studies to be conducted in order to trace the causes of CKDu in AP. This includes, but is not limited to, large study population, robust sampling, and extensive screening for comprehensive panels of chemicals in blood and/or urine. Until his happens, there is an immediate need for public health intervention to halt the spread of CKD in endemic areas like AP through strong enforcement of the use of potentially nephrotoxic chemicals, comprehensive and regular analysis of drinking water, and raising awareness about the use of personal protective equipment.

## Abbreviations

AP: Andhra Pradesh
BMI: body mass index
CKD: chronic kidney disease
CKDu: CKD of unknown etiology
eGFR: estimated glomerular filtration rate
IRB: Institutional Review Board
KDIGO: Kidney Disease Improving Global Outcomes
NABL: National Accreditation Board for Testing and Calibration Laboratories
PR: prevalence ratio
SD: standard deviations
SEEK: Screening and Early Evaluation of Kidney Disease

## References

[1] Ruggenenti P, Schieppati A, Remuzzi G. Progression, remission, regression of chronic renal diseases. Lancet. 2001;357(9268):1601–1608. 11377666 10.1016/S0140-6736(00)04728-0

[2] Wild S, Roglic G, Green A, et al. Global prevalence of diabetes: estimates for the year 2000 and projections for 2030. Diabetes Care. 2004;27(5):1047–1053. 15111519 10.2337/diacare.27.5.1047

[3] Singh AK, Farag YM, Mittal BV, et al. Epidemiology and risk factors of chronic kidney disease in India – results from the SEEK (Screening and Early Evaluation of Kidney Disease) study. BMC Nephrol. 2013;14:114. 23714169 10.1186/1471-2369-14-114 PMC3848478

[4] Gracia-Trabanino R, Dominguez J, Jansa JM, et al. Proteinuria and chronic renal failure in the coast of El Salvador: detection with low cost methods and associated factors. Nefrologia. 2005;25(1):31–38. 15789534

[5] Peraza S, Wesseling C, Aragon A, et al. Decreased kidney function among agricultural workers in El Salvador. Am J Kidney Dis. 2012;59(4):531–540. 22300650 10.1053/j.ajkd.2011.11.039

[6] Trabanino RG, Aguilar R, Silva CR, et al. End-stage renal disease among patients in a referral hospital in El Salvador. Rev Panam Salud Publica. 2002;12(3):202–206. 12396639 10.1590/s1020-49892002000900009

[7] Lebov JF, Valladares E, Pena R, et al. A population-based study of prevalence and risk factors of chronic kidney disease in León, Nicaragua. Can J Kidney Health Dis. 2015;2:6. 25926994 10.1186/s40697-015-0041-1 PMC4414463

[8] Torres C, Aragon A, Gonzalez M, et al. Decreased kidney function of unknown cause in Nicaragua: a community-based survey. Am J Kidney Dis. 2010;55(3):485–496. 20116154 10.1053/j.ajkd.2009.12.012

[9] Athuraliya NT, Abeysekera TD, Amerasinghe PH, et al. Uncertain etiologies of proteinuric-chronic kidney disease in rural Sri Lanka. Kidney Int. 2011;80(11):1212–1221. 21832982 10.1038/ki.2011.258

[10] Wanigasuriya KP, Peiris-John RJ, Wickremasinghe R, et al. Chronic renal failure in North Central Province of Sri Lanka: an environmentally induced disease. Trans R Soc Trop Med Hyg. 2007;101(10):1013–1017. 17643458 10.1016/j.trstmh.2007.05.006

[11] El Minshawy O. End-stage renal disease in the El-Minia Governorate, upper Egypt: an epidemiological study. Saudi J Kidney Dis Transpl. 2011;22(5):1048–1054. 21912050

[12] Cucuianu MP, Rus HG, Roman S, et al. Tissue-type plasminogen activator (t-PA) and dilute blood clot lysis time in nephrotic patients. Thromb Haemost. 1989;61(2):270–274. 2501897

[13] Levey AS, Stevens LA, Schmid CH, et al. A new equation to estimate glomerular filtration rate. Ann Intern Med. 2009;150(9):604–612. 19414839 10.7326/0003-4819-150-9-200905050-00006 PMC2763564

[14] Lee J. Odds ratio or relative risk for cross-sectional data? Int J Epidemiol. 1994;23(1):201–203. 8194918 10.1093/ije/23.1.201

[15] Machiraju RY, Gowrishankar S, Edwards KL, et al. Epidemiology of Udhanam endemic nephropathy. J Am Soc Nephrol. 2009;20:643A.

[16] Almaguer M, Herrera R, Orantes CM. Chronic kidney disease of unknown etiology in agricultural communities. MEDICC Rev. 2014;16(2):9–15. 24878644 10.37757/MR2014.V16.N2.3

[17] Tatapudi RR, Rentala S, Gullipalli P, et al. High prevalence of CKD of unknown etiology in Uddanam, India. Kidney Int Rep. 2019;4(3):380–389. 30899865 10.1016/j.ekir.2018.10.006 PMC6409405

[18] Rajapurkar MM, John GT, Kirpalani AL, et al. What do we know about chronic kidney disease in India: first report of the Indian CKD registry. BMC Nephrol. 2012;13:10. 22390203 10.1186/1471-2369-13-10 PMC3350459

[19] Trivedi H, Vanikar A, Patel H, et al. High prevalence of chronic kidney disease in a semi-urban population of Western India. Clin Kidney J. 2016;9(3):438–443. 27274831 10.1093/ckj/sfw009 PMC4886905

[20] Anand S, Shivashankar R, Ali MK, et al. Prevalence of chronic kidney disease in two major Indian cities and projections for associated cardiovascular disease. Kidney Int. 2015;88(1):178–185. 25786102 10.1038/ki.2015.58 PMC4490055

[21] Lunyera J, Mohottige D, Von Isenburg M, et al. CKD of uncertain etiology: a systematic review. Clin J Am Soc Nephrol. 2016;11(3):379–385. 26712810 10.2215/CJN.07500715 PMC4791820

[22] Weaver VM, Fadrowski JJ, Jaar BG. Global dimensions of chronic kidney disease of unknown etiology (CKDu): a modern era environmental and/or occupational nephropathy? BMC Nephrol. 2015;16:145. 26282933 10.1186/s12882-015-0105-6 PMC4539684

[23] Mohineesh RJ, Rajvanshi AC, Dogra TD, et al. Effect of acute exposure of triazophos on oxidative stress and histopathological alterations in liver, kidney and brain of Wistar rats. Indian J Exp Biol. 2014;52(8):814–819. 25141545

[24] Vikrant S. Hepato-renal toxicity-associated with methyl parathion exposure. Ren Fail. 2015;37(2):355–356. 25410113 10.3109/0886022X.2014.986620

[25] Du L, Li S, Qi L, et al. Metabonomic analysis of the joint toxic action of long-term low-level exposure to a mixture of four organophosphate pesticides in rat plasma. Mol Biosyst. 2014;10(5):1153–1161. 24626741 10.1039/c4mb00044g

[26] Larsen K, Najle R, Lifschitz A, et al. Effects of sub-lethal exposure of rats to the herbicide glyphosate in drinking water: glutathione transferase enzyme activities, levels of reduced glutathione and lipid peroxidation in liver, kidneys and small intestine. Environ Toxicol Pharmacol. 2012;34(3):811–818. 23044091 10.1016/j.etap.2012.09.005

[27] Guo J, Shi T, Cui X, et al. Effects of silica exposure on the cardiac and renal inflammatory and fibrotic response and the antagonistic role of interleukin-1 beta in C57BL/6 mice. Arch Toxicol. 2016;90(2):247–258. 25388157 10.1007/s00204-014-1405-5

[28] Vupputuri S, Parks CG, Nylander-French LA, et al. Occupational silica exposure and chronic kidney disease. Ren Fail. 2012;34(1):40–46. 22032652 10.3109/0886022X.2011.623496 PMC3266824

[29] Mascarenhas S, Mutnuri S, Ganguly A. Deleterious role of trace elements – silica and lead in the development of chronic kidney disease. Chemosphere. 2017;177:239–249. 28292724 10.1016/j.chemosphere.2017.02.155

[30] Reddy DV, Gunasekar A. Chronic kidney disease in two coastal districts of Andhra Pradesh, India: role of drinking water. Environ Geochem Health. 2013;35(4):439–454. 23475496 10.1007/s10653-012-9506-7

